# Incidental Finding of Strut Malapposition Is a Predictor of Late and Very Late Thrombosis in Coronary Bioresorbable Scaffolds

**DOI:** 10.3390/jcm8050580

**Published:** 2019-04-27

**Authors:** Niklas F. Boeder, Melissa Weissner, Florian Blachutzik, Helen Ullrich, Remzi Anadol, Monique Tröbs, Thomas Münzel, Christian W. Hamm, Jouke Dijkstra, Stephan Achenbach, Holger M. Nef, Tommaso Gori

**Affiliations:** 1 Medical Clinic I, University Hospital of Giessen, Klinikstrasse 33, 35392 Giessen, Germany; niklas.boeder@innere.med.uni-giessen.de (N.F.B.); Florian.Blachutzik@innere.med.uni-giessen.de (F.B.); christian.hamm@innere.med.uni-giessen.de (C.W.H.); holger.nef@innere.med.uni-giessen.de (H.M.N.); 2 Zentrum für Kardiologie, University Hospital Mainz, Langenbeckstrasse 1, 55131 Mainz, Germany and German Center for Cardiac and Vascular Research (DZHK), Standort Rhein-Main; melissaweissner@web.de (M.W.); hullrich@students.uni-mainz.de (H.U.); remzi.anadol@unimedizin-mainz.de (R.A.); tmuenzel@uni-mainz.de (T.M.); 3 Department of Cardiology, University Hospital of Erlangen, Ulmenweg 18, 91054 Erlangen, Germany; monique.troebs@uk-erlangen.de (M.T.); stephan.achenbach@uk-erlangen.de (S.A.); 4 Department of Radiology, Leiden University Medical Center, P.O. Box 9600 (mailstop C2-S), 2300 Leiden, The Netherlands; j.dijkstra@lumc.nl

**Keywords:** stent thrombosis, bioresorbable scaffold, optical coherence tomography

## Abstract

Malapposition is a common finding in stent and scaffold thrombosis (ScT). Evidence from studies with prospective follow-up, however, is scarce. We hypothesized that incidental observations of strut malapposition might be predictive of late ScT during subsequent follow-up. One hundred ninety-seven patients were enrolled in a multicentre registry with prospective follow-up. Optical coherence tomography (OCT), performed in an elective setting, was available in all at 353 (0–376) days after bioresorbable scaffold (BRS) implantation. Forty-four patients showed evidence of malapposition that was deemed not worthy of intervention. Malapposition was not associated with any clinical or procedural parameter except for a higher implantation pressure (*p* = 0.0008). OCT revealed that malapposition was associated with larger vessel size, less eccentricity (all *p* < 0.01), and a tendency for more uncovered struts (*p* = 0.06). Late or very late ScT was recorded in seven of these patients 293 (38–579) days after OCT. OCT-diagnosed malapposition was a predictor of late and very late scaffold thrombosis (*p* < 0.001) that was independent of the timing of diagnosis. We provide evidence that an incidental finding of malapposition—regardless of the timing of diagnosis of the malapposition—during an elective exam is a predictor of late and very late ScT. Our data provide a rationale to consider prolonged dual antiplatelet therapy if strut malapposition is observed.

## 1. Introduction

Bioresorbable scaffolds (BRS) were introduced to offer transient vessel support after coronary angioplasty while avoiding long-term risks associated with permanent metallic stents [1]. However, the BRS with by far the most clinical experience, the everolimus-eluting Absorb BRS (Abbott Vascular, Santa Clara, CA, USA), showed an unexpectedly high incidence of scaffold thrombosis (ScT) both early and late after implantation in a number of single- and multicentre observational studies [2,3,4,5,6] and was ultimately removed from the market. Incomplete expansion of the BRS is believed to convey the highest risk of early ScT and implantation techniques aimed to achieve full expansion of the device were shown to reduce the incidence of early ScT [7]. In contrast, studies based on quantitative coronary angiography provided evidence that undersizing (i.e., choice of a BRS smaller than the reference vessel) is strongly associated with late adverse events [8,9]. The mechanism(s) of this association remain speculative, however, it can be hypothesized that malapposition and the resulting disturbances in blood flow dynamics might play a role.

While the mechanistic rationale for the association of malapposition and late stent/scaffold thrombosis is solid [10,11], evidence to date is limited to observational studies in which malapposition was frequently found in cases of ScT [12,13,14,15,16]. Therefore, the aim of this study was to investigate whether incidental observation of scaffold malapposition would predict subsequent late and very late ScT.

## 2. Methods

### 2.1. Objective of the Study

The hypothesis of the study was that incidental optical coherence tomography (OCT) evidence of scaffold malapposition (observed in an elective setting and not deemed worthy of intervention at the time of OCT) might predict the occurrence of ScT during subsequent follow-up.

### 2.2. Patients

Consecutive patients treated at three high-volume centres in Germany (University of Mainz; University of Giessen; University of Erlangen) with Absorb BRS between April 2012 and March 2016 who satisfied the following criteria were included in this multicentre registry:

- Patients had undergone elective OCT at the end of the implantation procedure, during non-target vessel-staged procedures, or in the setting of elective invasive exams.
- In none of these patients was there evidence of ischemia in the region perfused by the vessel treated with BRS and OCTs had been performed as elective controls after implantation of these novel devices.
- An experienced interventionalist (based on current experts´ recommendations [17]) reviewed the OCT and saw no clinical indication for the re-treatment of these lesions.

Patient and procedural data were entered retrospectively after inclusion in the study. Follow-up data were acquired prospectively by trained medical staff during clinical visits and telephone interviews. Referring cardiologists, general practitioners, and patients were contacted whenever necessary for further information. All data were internally audited at each centre by trained local staff and were entered retrospectively into the multicentre database in an anonymized way according to national privacy policies and laws and following the requirements of the local ethics committees. Data were audited centrally for consistency and plausibility and queries were generated when necessary.

### 2.3. Definitions

Frequency domain-OCT was performed using the Ilumien Optis system (St. Jude Medical, Inc., Minneapolis, MN, USA). OCT imaging catheters were inserted distally to the treated segments and the pullback was recorded until either the guiding catheter was reached or the maximum pullback length was completed. If necessary, two sequential pullbacks were acquired to image the scaffolded segment.

OCT measurements were made offline using the QCU-CMS software (Medis, Leiden, Netherlands) by trained staff using standardized operating procedures. Longitudinal cross-sections were analysed at 1 mm intervals within the stented lesion and 5 mm proximally and distally to the scaffold. Among others, the following quantitative parameters were determined: The percentage of incomplete strut apposition (ISA) at 1 mm intervals calculated as a percentage of the total number of malapposed struts divided by the total number of struts; malapposition distance, length, and area; the eccentricity index computed as the ratio between the minimum and maximum diameters; the symmetry index defined as the difference between maximum scaffold diameter and minimum scaffold diameter divided by the maximum scaffold diameter; presence of evaginations, peri-strut low intensity areas (PSLIA), and microvessels. Evaginations were defined as any outwards protrusion in the luminal vessel contour beyond the struts´ abluminal surface between well-apposed struts. Strut discontinuity/disruptions were diagnosed if there was evidence of isolated (malapposed) struts or groups of struts that did not fit the normal circular geometry of the scaffold in one or more than one cross section by more than 33% of the distance between the centre of gravity and the lumen. Further, cases where there was evidence of a clear gap (frames without any strut) were also diagnosed as strut discontinuity [18].

Definitions are described in detail in [19]. Briefly, malapposition was defined as a lack of contact of at least 1 strut with the underlying vessel wall (at least 150 μm, in the absence of a side branch) with evidence of blood flow behind the strut. It was classified as “major” malapposition if there was evidence of at least 30% of the struts in one frame. Neovessels were defined as sharply delimitated, signal-poor lacunae that extended over multiple contiguous frames. Peri-strut low intensity areas (PSLIAs) were defined as homogeneous, non-signal-attenuating zones around struts that were of lower intensity than the surrounding tissue. Peri-strut intensity was measured at the mid-strut at a depth of 150 μm from the lumen and at equal distance between two contiguous struts based on intensity of the “key” component of the CMYK (cyan, magenta, yellow, and key) colour model based on raw cross-sectional images. Quantitative assessment was obtained at 5 mm proximal and distal to the BRS to measure the proximal and distal reference vessel area (RVA). RVA was calculated as the mean of the 2 largest luminal areas 5 mm proximal and distal to the BRS edge [20]. If no meaningful value for proximal or distal RVA was obtained, the largest luminal cross-sectional area at either end was used. Incomplete expansion was defined as a minimum scaffold area of at least 90% in both the proximal and distal halves of the scaffold relative to the closest reference segment.

ScT was centrally adjudicated and classified as definite, probable, and possible according to the Academic Research Consortium criteria based on the analysis of original documents [21]. Definite ScT required angiographic or autopsy confirmation with thrombus originating in the BRS or in the segment 5mm proximal or distal to the BRS. Probable ScT was considered to have occurred after intracoronary stenting in the following cases: any unexplained death within the first 30 days or any myocardial infarction—irrespective of the time after the index procedure—that is related to documented acute ischemia in the region of the implanted BRS without angiographic confirmation, in the absence of any other obvious cause. Possible ScT was considered to have occurred with any unexplained death from 30 days after intracoronary stenting until the end of trial follow-up.

### 2.4. Statistical Analysis

Statistical analysis was performed using IBM SPSS Statistics (SPSS Statistics 23, IBM Deutschland GmbH, Ehningen, Germany). Categorical data are presented as absolute numbers and percentages. Continuous variables are given as mean (SD) or median (IQR). The frequencies of categorical variables were compared by the Pearson chi-square test and the distribution of continuous variables was compared by the Mann–Whitney–Wilcoxon test. No imputation was performed. A Kaplan–Meier curve was used to plot time-to-event curves and the hypothesis that malapposition could be associated with incident ScT was tested using a log-rank test. Exploratory univariate and multivariable Cox regression analysis was performed to evaluate the impact of each of the above parameters on the occurrence of ScT. Potential covariates were prioritized for data analysis (a list of the covariates is presented in Supplementary Tables S4–S6). To address the impact of the timing of the OCT diagnosis on the association between malapposition and ScT, the period until diagnosis of malapposition was grouped into early (diagnosis within 48 h after implantation of BRS), mid (diagnosis from day 3 but not later than 30 days after implantation of BRS), and late (later than 30 days after the implantation of BRS). This variable was also entered into the Cox model. The threshold for statistical significance was *p* < 0.05.

## 3. Results

### 3.1. Patient Characteristics

A total of 197 patients (219 lesions) who underwent elective OCT within 353 (0–376) days after BRS implantation were enrolled in the study (Table 1). One hundred and thirty-two patients were treated in Mainz, 36 in Giessen, and 29 in Erlangen. Follow-up was complete (100%) at a median of 1059 (1009–1110) days, during which 7 patients presented with late or very late ScT 579 [341–623] days after implantation and 293 (38–579) days after OCT. Diagnosis of ScT was supported by OCT-imaging in 5 cases and was based on angiography alone in two patients.

Patients with late or very late ScT showed similar characteristics with respect to age, sex, and cardiovascular risk profile. A history of prior revascularization was more frequent in ScT patients (71.4% vs. 34.7%; *p* = 0.04). The majority of BRS were implanted in the setting of an acute coronary syndrome (71.4% vs. 51.4%; *p* = 0.30) with no difference between the groups. While control patients tended to have a higher number of scaffolds per lesion (1.0 ± 0 vs. 1.2 ± 0.5; *p* = 0.25), the number of treated vessels (1.6 ± 0.8 vs. 1.1 ± 0.4; *p* = 0.01) was higher in the ScT group. Parameters of lesion complexity were comparable between the groups. 

Procedural characteristics divided by the incidence of ScT are shown in Table S1. The strategy used for implantation was not different between ScT patients and reference patients; pre-dilatation was performed in almost all cases (100% vs. 99.5%; *p* = 0.85) with comparable inflation pressures (13.4 ± 1.9 vs. 13.2 ± 2.3 atm; *p* = 0.75). All ScTs (except for one, occurring during clopidogrel therapy at 38 days after index) were observed after scheduled cessation of dual antiplatelet therapy (DAPT).

### 3.2. Optical Coherence Tomography (OCT) Characteristics

**Figure 1:**
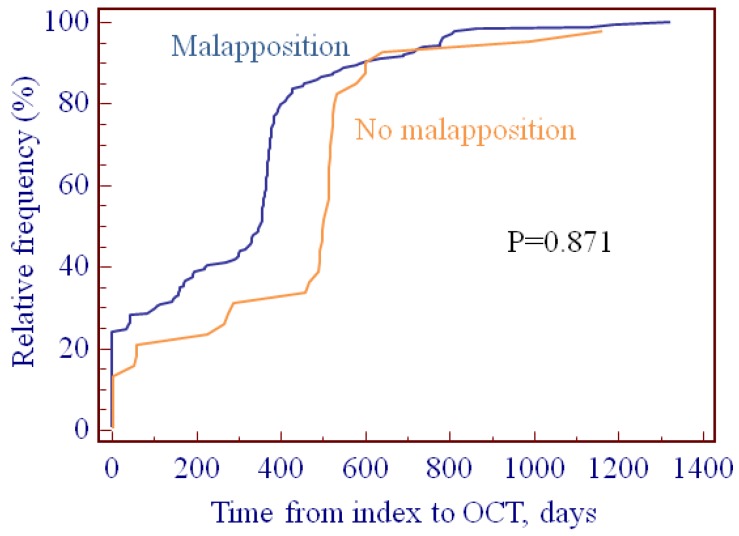
Timing of OCT from index procedure. There was no difference between patients with or without malapposition (*p* = 0.871).

The timing of OCT from the index procedure is presented in Figure 1. OCT characteristics are shown in Table 2. Total scaffold length did not differ significantly between ScT patients and reference patients (35.4 ± 29.2 vs. 27.4 ± 16.5 mm; *p* = 0.96). At the patient level, there was no difference in scaffold nominal diameter, however, at lesion level, scaffolds that displayed an ScT during follow-up had a greater minimum (3.2 ± 0.22 vs. 3.0 ± 0.35 mm; *p* = 0.01) and maximum scaffold diameter (3.4 ± 0.24 vs. 3.1 ± 0.34 mm; *p* = 0.04). In line with this, the maximum (12.3 ± 2.5 vs. 9.1 ± 3.1 mm^2^; *p* = 0.005) and minimum (6.3 ± 1.0 vs. 4.8 ± 1.9 mm^2^; *p* = 0.82) lumen areas were significantly larger in ScT patients. The incidence of PSLIA and neovessels as well as BRS asymmetry and eccentricity were similar between ScT and reference patients (Table 2). Strut discontinuities were more frequently observed in the ScT patients (42.9% vs. 5.9%; *p* < 0.001). Furthermore, uncovered (42.9% vs. 5.9%; *p* < 0.001) and malapposed struts (85.7% vs. 20.1%; *p* < 0.001) were observed significantly more frequently in BRS that later developed ScT. Malapposition area, distance, and length were not different in patients with compared with those without ScT.

Baseline characteristics and procedure-related parameters of patients with and without malapposition can be found in Tables S2 and S3 (supplementary materials). The presence of malapposition was not associated with any of the other OCT characteristics. Patients with malapposition, however, showed a larger lumen area (8.4 ± 2.5 vs. 11.6 ± 3.8 mm; *p* < 0.001) and more eccentricity (0.68 ± 0.09 vs. 0.61 ± 0.12; *p* < 0.001) and tended to have uncovered struts more frequently (*p* = 0.06, Table S8, supplementary materials).

### 3.3. Analysis of the OCT Predictors of Scaffold Thrombosis

**Figure 2:**
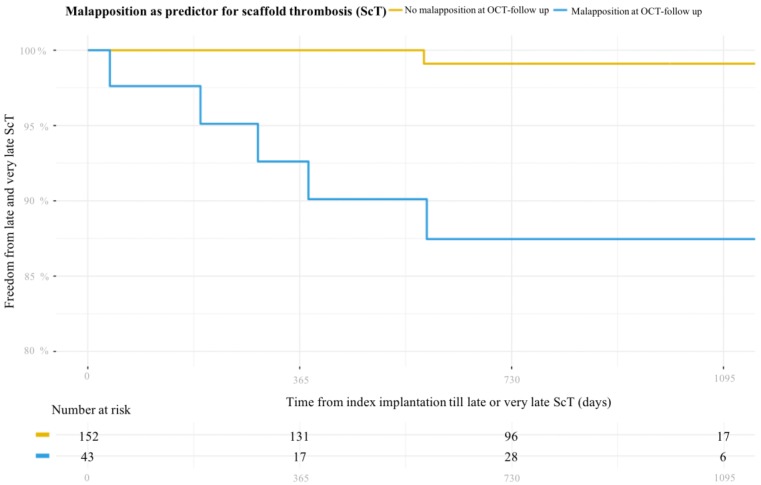
Kaplan–Meier curve illustrating the association between incidental finding of malapposition during elective follow-up OCT and incidence of late and very late ScT.

Figure 2 illustrates the relationship between OCT evidence of malapposition and subsequent ScT. The log rank test *p* was <0.001.

Multivariable Cox regression identified both malapposition diagnosed early (within 48 hours) after the index procedure (HR = 24.1, 95% CI = 1.5–387.6, *p* = 0.03) and later than day 30 (HR = 20.9, 95% CI = 2.5–179.7, *p* < 0.001) as independent predictors of ScT. Further exploratory univariate and multivariable Cox regression analysis (Tables S4–S7, supplementary materials) showed that the presence of malapposition (*p* = 0.049, hazard ratio (HR) 10.56 (1.0–110.68)) was the only independent predictor of ScT. The presence of uncovered struts and the number of vessels treated showed a threshold association (*p* = 0.05).

### 3.4. OCT Evidence at the Time of ScT

OCT observations at the time of ScT were collected in a separate retrospective registry. These data are presented in Appendix A.

## 4. Discussion

The principal findings of this study are: (1) Malapposition was identified as predictor of late and very late ScT in patients treated with BRS; this association was demonstrated for both “major” malapposition (defined as evidence of at least 30% of the struts in one frame) and for any degree of malapposition. This association was valid when malapposition was diagnosed either at implantation or >30 days thereafter. (2) The presence of uncovered struts showed a threshold association with the incidence of ScT during follow-up in multivariate analysis. (3) In an analysis of thrombi detected by OCT (see supplementary materials), evidence of malapposition was more frequent in late/very late ScT than in early ScT. This evidence was associated with larger vessel and scaffold sizes.

BRS were introduced to overcome long-term limitations of metallic stents; however, evidence from a number of registries and randomized controlled studies showed BRS to be associated with increased risk of both early and late ScT [2,4,22]. Importantly, early ScT was often shown to be associated with procedural issues (including suboptimal vessel sizing and incomplete scaffold/vessel expansion) [23]. In line with this, improvement in implantation techniques proved to be associated with reduced rates of such events [24,25]. Later, evidence was reported of late and very late ScT, i.e., at a time when the benefits of the resorbable device over metallic stents were supposed to be realized [2,4,22]. Importantly, malapposition, often associated with strut discontinuity and uncovered struts, emerged as the strongest association of very late ScT in OCT case series [13,15,16]. Although the mechanisms of this form of disruption in the geometry of the scaffold remain unknown and are probably different from case to case, the persistence of early malapposed (often uncovered) struts, or the development of late malapposition/evaginations, both resulting in struts not being embedded in the vascular wall, appear to be a prerequisite for evolution of adverse scaffold geometry. Similar data are also available for metallic stents; malapposition was the leading finding in the Bern and PESTO registries of stent thrombosis and among the three leading mechanisms in the PRESTIGE registry [26,27].

Stent malapposition results in disturbances in blood flow dynamics, adluminal areas of high shear stress, and abluminal areas of low shear stress and recirculation [10,11]. These disturbances are in turn associated with impaired strut coverage by endothelial cells (“strut healing“), strongly influence the local levels of blood viscosity, and stimulate platelet activation and neointima formation [28,29] that might amplify the supposedly higher thrombogenicity of scaffolds [30].

Despite this rationale and retrospective evidence, prospective follow-up data on the role of malapposition in determining an increased risk of late thrombosis in BRS remain limited to first-generation drug-eluting stents, in which the occurrence of late acquired malapposition at any time between imaging assessment and stent thrombosis complicates any assessment. In a meta-analysis by Hassan et al, the odds ratio for the risk of stent thrombosis in patients with diagnosed late acquired malapposition was 6.51 (1.34–34.91); however, the data were heterogeneous, with three trials supporting this conclusion and the other two leaning in the other direction. These considerations have a further level of complexity with respect to BRS use, where it would be expected that the resorption of the malapposed struts would limit their thrombogenic potential. Gomez-Lara et al. [31] performed an OCT sub-study of the ABSORB trial Cohort B, in which BRS having a 3 mm diameter were deployed. The incidence of malapposed struts in vessels with a final distal maximal lumen diameter >3.3 mm was higher than in cases in which the final lumen diameter was smaller. In addition, we previously reported that undersizing at the time of implantation and BRS implantation in vessels larger than 3.5 mm are predictors of late/very late ScT. In contrast, oversizing and small reference vessel diameters (RVDs) were predictors of early ScT [18]. The current data provide a possible mechanism related to this observation, suggesting that malapposition, even when it is diagnosed incidentally and regardless of the length of time from implantation, is a predictor of late and very late ScT and should therefore trigger mechanical/pharmacological intervention.

In our database, total scaffold length, uncovered struts, minimum lumen area, and the number of vessels treated did not affect the chances of ScT in our cohort, although the regression model should be analyzed with caution as the covariate/case ratio may have led to overmodeling [32].

## 5. Limitations

There are several limitations associated with this study. First, its registry nature, with retrospective collection of patients´ data and prospective follow-up, has clear inherent limitations, and the evidence provided here (particularly given the small sample size and event rate) should be seen as hypothesis generating, particularly with regard to the exploratory multivariate analysis. A strictly prospective study design to investigate malapposition as a causal factor of scaffold thrombosis would be complex from an ethical perspective. OCT follow-up was not performed at fixed time points and was not repeated, so any conclusion on the nature (early versus late acquired) of malapposition was impossible. Further, the Bern registry emphasized the importance of the longitudinal extent of malapposition (>1 mm) and suggested a cut-off >300 μm for the strut-vessel wall distance [33]. These cases are usually treated in the clinical routine and were not included in the present database. The present study expands this evidence to suggest that findings of scaffold malapposition, even those that appear as “minor” and not worthy of intervention, are indeed associated with ScT. Importantly, fluid dynamic models demonstrate that, particularly for thick struts with quadratic profile, a smaller malapposition distance might actually have the largest hemodynamic effect [28,29]. ScT is a complex phenomenon in which vessel, scaffold architecture, structure, and a number of patient characteristics play a role and it is likely that larger cohorts would have allowed identification of other clinical or procedural parameters and possible causes and mechanisms. For our analysis, late and very late ScT were pooled. However, all late ScT occurred at a time at which resorption would have already started and the two groups of patients did not differ in any of the key features. The definition of strut fracture has not yet been validated and an analysis of different types of fractures goes beyond the scope of this study. We therefore limited the definition of fracture to cases where discontinuity, altered geometry (see example in the supplement data), and/or a gap within the scaffold was evident. Our data apply to “thick strut” scaffolds; no conclusion on the importance of malapposition in (thin-strut) metallic stents can be inferred. Finally, the cross-sectional nature of our observation does not allow mechanistic insight. The (non-significantly) higher incidence of ST-segment elevation myocardial infarctions (STEMIs) at index in the patients presenting with malapposition points out the importance (and complexity) of vessel sizing in this setting.

## 6. Conclusions

We provide the first evidence that an incidental finding of malapposition—regardless of the timing of diagnosis of the malapposition—during an elective exam is a predictor of late and very late ScT. Whether mechanical correction of this finding also reduces events is unknown and will require further studies. However, this evidence suggests that prolonging dual antiplatelet therapy in these cases would be a prudent strategy.

## Figures and Tables

**Table 1:** Baseline characteristics of the cohort.

Baseline Characteristic	Late or Very Late Scaffold Thrombosis ScT (*n* = 7)	No ScT (*n* = 190)	*p*
Age (years)	58.3 ± 9.1	61.8 ± 11.9	0.37
Male Sex (%)	85.7	81.6	0.78
Hypertension (%)	100	77.9	0.16
Diabetes mellitus (%)	14.3	20.5	0.69
Current smoker (%)	43.9	36.8	0.74
Family history (%)	14.3	30.0	0.37
Hyperlipoproteinaemia (%)	43.9	47.4	0.81
Prior revascularization (%)	71.4	34.7	0.04 *
Prior bypass surgery (%)	0	3.7	0.06
Prior percutaneous intervention (%)	71.4	33.2	0.04 *
Prior stroke/TIA (%)	0	3.2	0.63
eGFR (mean ± SD, ml/min)	91.4 ± 30.8	85.4 ± 20.2	0.74
Left ventricular ejection fraction (mean ± SD, %)	54.3 ± 7.9	54 ± 8.2	0.96
Acute coronary syndrome (%)	71.4	51.4	0.30
Clinical indication			
- Stable angina (%)	28.6	37.4	0.57
- ST-elevation myocardial infarction (%)	42.9	22.1	0.29
- Non-ST-elevation myocardial infarction (%)	28.6	24.9	0.82
- Unstable angina (%)	0	13.8	0.20
Number of vessels treated	1.6 ± 0.8	1.1 ± 0.4	0.01 *
Number of scaffolds per lesion	1.0 ± 0	1.2 ± 0.5	0.25
Number of scaffolds per patient	1.7 ± 1.1	1.3 ± 0.7	0.23
Chronic total occlusion (%)	0	6.8	0.47
Lesion type AHA/ACC classification B/C2 (%)	85.7	63.5	0.22
Dual antiplatelet therapy (DAPT)			0.58
- Clopidogrel (%)	14.3	31.2	
- Prasugrel (%)	14.3	52.4	
- Ticagrelor (%)	71.4	16.4	

* = statistically significant; eGFR = estimated Glomerular filtration rate; AHA/ACC = American Heart Association/American College of Cardiology.

**Table 2:** Optical coherence tomography (OCT) findings.

Optical Coherence Finding	Late or Very Late ScT (*n* = 7)	No ScT (*n* = 190)	*p*
Number of struts	1080 ± 485	1059 ± 837	0.38
Number of frames	116.1 ± 84	120.4 ± 49	0.24
Pullback length (mm)	19.1 ± 8.1	21.1 ± 5.4	0.35
Maximum lumen area (mm^2^)	12.3 ± 2.5	9.1 ± 3.1	0.005 *
Minimum lumen area (mm^2^)	6.3 ± 1.0	4.8 ± 1.9	0.02 *
Average lumen area (mm^2^)	8.96 ± 1.03	6.6 ± 2.20	0.003 *
Maximum lumen asymmetry	0.28 ± 0.10	0.27 ± 0.11	0.82
Maximum scaffold asymmetry	0.24 ± 0.012	0.24 ± 0.09	0.91
Maximum lumen eccentricity	0.62 ± 0.11	0.66 ± 0.10	0.29
Maximum scaffold eccentricity	0.66 ± 0.10	0.72 ± 0.08	0.07
Peri-strut low intensity area (PSLIA) (%)	20.0	5.4	0.18
Microvessels (%)	42.9	31.0	0.51
Fractures (%)	57.1	33.5	0.20
Uncovered scaffold struts (%)	42.9	5.8	<0.001 *
Malapposition (>30% in one frame, without side) (%)	71.4	15.3	<0.001 *
Any malapposition per patient (%)	85.7	20.1	<0.001 *
Malapposition length (mm)	2.33 ± 1.5	2.76 ± 1.8	0.67
Malapposition maximum area (mm^2^)	1.56 ± 0.69	2.3 ± 1.9	0.64
Number of malapposed segments	1.83 ± 1.17	1.7 ± 0.99	0.88
Malapposition distance (mm)	0.52 ± 0.25	0.89 ± 0.77	0.22
Evagination (%)	57.1	27.5	0.08

* = statistically significant.

**Figure A1:**
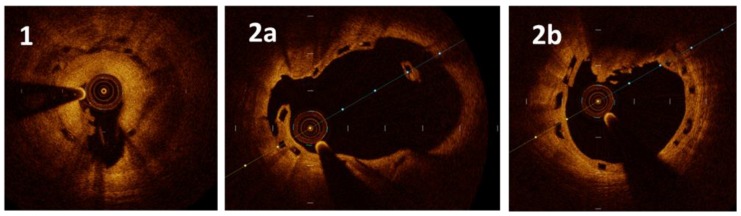
Representative images of cases of late/very late scaffold thrombosis (ScT) (*n* = 9). The panels are numbered according to the patient number. Case **1**: ScT 563 days after index. The patient presented with ST-segment elevation myocardial infarction (STEMI). Optical coherence tomography (OCT) shows a homogeneous layer within the bioresorbable scaffold (BRS), suggestive of neointima, evidence of white thrombus (7 o´clock), and a large signal-poor, peri-strut, low intensity area (from 1 to 5 o´clock) with increased attenuation compatible with oedema/immature neointima. The architecture of the struts appears to be disrupted, but no gap was evident. Case **2**: ScT 349 days after index. The patient presented with non-ST-segment elevation myocardial infarction (NSTEMI). OCT revealed malapposition (**2a**) immediately contiguous to white-red thrombus (**2b**). In this case, the strut architecture was also altered (**2b**; 1 o´clock) and there was evidence of incompletely covered struts (**2a**).

## Supplementary Materials

The following are available online at https://www.mdpi.com/2077-0383/8/5/580/s1, Table S1: Procedural characteristics of the prospective follow-up observational cohort, Table S2: Baseline characteristics of patients depending on presence of malapposition, Table S3: Procedural characteristics depending on presence of malapposition, Table S4: Univariate analysis of baseline characteristics for primary endpoint (late or very late ScT), Table S5: Univariate analysis of procedural characteristics for primary endpoint (late or very late ScT), Table S6: Univariate analyse of OCT findings for primary endpoint (late or very late ScT), Table S7: Multivariable Cox regression analysis for primary endpoint (late or very late ScT), Table S8: OCT findings depending on presence of malapposition, Table S9: Baseline characteristics of patients with ScT, Table S10: Procedural characteristics in the retrospective cohort of patients with ScT, Table S11: OCT findings in the retrospective cohort (OCT at the time of ScT), Table S12: Dual antiplatelet regime in the cases in which OCT was diagnosed at the time of ScT.

## Appendix A

### A.1. Retrospective Cohort: Malapposition at the Time of Scaffold Thrombosis (ScT)

Optical coherence tomography (OCT) recordings of consecutive patients with ScT were collected in an additional retrospective registry study (Figure A1). Observations in cases of late/very late ScT were compared with those in patients with early ScT.

### A.2. Results: OCT Evidence at the Time of ScT

All patients presenting with ScT in whom an OCT was performed at the three institutions were included in a separate retrospective registry. A total of 16 patients with OCT acquisition at the time of the ScT were identified. Five of these patients were also included in the prospective follow-up observational group (31.3%). Seven patients of the retrospective cohort were classified as acute or subacute and nine as late or very late ScT (563 (49) days after implantation). Representative images of the thrombi are presented in Figure A1. All ScTs were classified as definite. Patients with late or very late ScT were more frequently female and more frequently had a history of prior revascularization. Further baseline and procedural characteristics were similar (Tables S9 and S10, supplementary materials).

OCT findings are listed in Table S11 (supplementary materials). The majority of frames showed evidence of thrombus (78 ± 37 vs. 55% ± 66%; *p* = 0.18). Incomplete expansion was seen predominantly in cases of early ScT (83.3% vs. 12.4%; *p* = 0.008). Scaffold discontinuities (0% vs. 50%; *p* = 0.04) and malapposition (16.7% vs. 87.5%; *p* = 0.008) were features of late or very late ScT. OCT parameters expressing vessel and scaffold geometry did not differ between early and late ScT (Table S11, supplementary materials). The initial dual antiplatelet therapy (DAPT) regimen in use when implanting bioresorbable scaffold (BRS) was also not different (*p* = 0.66); however, 83% of patients developed late or very late ScT while not on DAPT (*p* = 0.006 compared to early ScT cases, Table S12, supplementary materials).
