# Supplementary material for: Incidental Finding of Strut Malapposition Is a Predictor of Late and Very Late Thrombosis in Coronary Bioresorbable Scaffolds
Source: J Clin Med. 2019 Apr 27;8(5):580. doi: 10.3390/jcm8050580 (PMC6571797; doi:10.3390/jcm8050580)
Supplement: Supplementary file 1 [file jcm-08-00580-s001.pdf]

Incidental Finding of Strut Malapposition is a Predictor of Late and Very Late Thrombosis in Coronary Bioresorbable Scaffolds

Table S1: Procedural characteristics of the prospective follow-up observational cohort.

	Late or very late ScT (n = 7)	No ScT (n = 212)	p
Pre-dilatation (%)	100	99.5	0.85
Pre-dilatation: Balloon diameter (mm)	2.9 ± 0.19	2.8 ± 0.35	0.19
Minimum scaffold diameter per lesion (mm)	3.2 ± 0.22	3.0 ± 0.35	0.01*
Minimum scaffold diameter per patient (mm)	3.0 ± 0.45	3.0 ± 0.35	0.70
Maximum scaffold diameter per lesion (mm)	3.4 ± 0.24	3.1 ± 0.34	0.04*
Total scaffold length (mm)	35.4 ± 29.2	27.4 ± 16.5	0.96
Maximum inflation pressure scaffold deployment (atm)	13.4 ± 1.9	13.2 ± 2.3	0.75
Post-dilatation in all scaffolds (%)	42.9	39.5	0.86
Post-dilatation: Balloon diameter (mm)	3.5 ± 0.5	3.3 ± 0.4	0.48
Post-dilatation: Maximum inflation pressure (atm)	12.7 ± 1.2	12.6 ± 2.5	0.59
Overlap (%)	0	14.2	0.28

Table S2: Baseline characteristics of patients depending on presence of malapposition.

	No Malapposition (n =153)	Malapposition (n = 44)	p
Age (years)	61.4 ± 11.3	62.3 ± 12.8	0.45
Male sex (%)	78.9	90.9	0.07
Hypertension (%)	78.3	79.5	0.80
Diabetes mellitus (%)	18.4	25.0	0.34
Current smoker (%)	37.5	36.4	0.89
Family history (%)	29.6	27.3	0.74
Hyperlipoproteinaemia (%)	49.3	38.6	0.12
Prior revascularization (%)	35.5	38.6	0.71
Prior percutaneous intervention (%)	34.2	36.4	0.79
Prior stroke/TIA (%)	2.6	4.5	0.51
eGFR (mean ± SD, ml/min)	84.9 ± 20.1	87.7 ± 21.7	0.42
Left ventricular ejection fraction (mean±SD, %)	54.1 ± 8.7	53.6 ± 6.0	0.27
Acute coronary syndrome (%)	50.0	58.1	0.35
Clinical indication			
Stable angina (%)	38.9	39.5	0.94
ST-elevation myocardial infarction (%)	22.2	25.6	0.65
Non-ST-elevation myocardial infarction (%)	27.1	18.6	0.26
Unstable angina (%)	11.8	16.3	0.44
Number of vessels treated	1.1 ± 0.34	1.3 ± 0.5	0.07
Number of scaffolds per lesion	1.2 ± 0.5	1.2 ± 0.4	0.93
Number of scaffolds per patient	1.3 ± 0.7	1.5 ± 0.8	0.19
Chronic total occlusion (%)	7.9	2.3	0.19
Lesion type	1.9	1.8	0.42
Dual antiplatelet therapy (DAPT)			0.58
Clopidogrel (%)	30.5	31.8	
Prasugrel (%)	51.7	56.8	
Ticagrelor (%)	17.9	11.4	

Table S3: Procedural characteristics depending on presence of malapposition.

	No malapposition (n = 153)	Malapposition (n = 44)	p
Pre-dilatation (%)	99.3	100	0.58
Pre-dilatation: Balloon diameter (mm)	2.8 ± 0.36	2.8 ± 0.30	0.23
Minimum scaffold diameter per lesion (mm)	3.0 ± 0.34	3.1 ± 0.37	0.27
Minimum scaffold diameter per patient (mm)	3.0 ± 0.34	3.0 ± 0.40	0.99
Maximum scaffold diameter per lesion (mm)	3.1 ± 0.33	3.1 ± 0.37	0.30
Total scaffold length (mm)	26.8 ± 16.2	30.1 ± 19.4	0.40
Maximum inflation pressure scaffold deployment (atm)	12.9 ± 2.2	14.1 ± 2.1	0.008
Post-dilatation in all scaffolds (%)	40.1	38.6	0.85
Post-dilatation: Balloon diameter (mm)	3.3 ± 0.3	3.3 ± 0.5	0.31
Post-dilatation: Maximum inflation pressure (atm)	12.7 ± 2.7	12.3 ± 0.7	0.97
Overlap (%)	12.5	15.9	0.56

Table S4: Univariate analysis of baseline characteristics for primary endpoint (late or very late ScT).

	p	Hazard ratio [95% CI]
Age (years)	0.43	0.98 [0.9–1.0]
Male sex (%)	0.72	1.5 [0.2–12.2]
Diabetes mellitus (%)	0.68	0.64 [0.1–5.3]
Current smoker (%)	0.70	1.3 [0.3–6.0]
Family history (%)	0.39	0.40 [0.04–3.3]
Hyperlipoproteinaemia (%)	0.90	0.91 [0.2–4.0]
Prior revascularization (%)	0.06	4.6 [0.9–24.5]
eGFR (mean±SD, ml/min)	0.50	1.0 [0.97–1.0]
Left ventricular ejection fraction (mean ± SD, %)	0.89	1.0 [0.91–1.1]
Acute coronary syndrome (%)	0.39	2,1 [0.4–10.7]
Clinical indication		
Stable angina (%)	0.60	0.65 [0.16–3.3]
ST-elevation myocardial infarction (%)	0.21	2.6 [0.6–11.6]
Non-ST-elevation myocardial infarction (%)	0.8	1.1 [0.2–6.2]
Number of vessels treated per patient	0.01*	3.6 [1.3–9.9]
Number of scaffolds per lesion	0.46	0.07 [0–73.9]
Number of scaffolds per patient	0.25	1.47 [0.77–2.8]
Lesion type	0.68	1.2 [0.6–2.3]
Dual antiplatelet therapy (DAPT)	0.56	1.3 [0.5–4.0]

Table S5: Univariate analysis of procedural characteristics for primary endpoint (late or very late ScT).

	p	Hazard ratio
Pre-dilatation: Balloon diameter (mm)	0.24	3.5 [0.43–27.9]
Minimum scaffold diameter per patient (mm)	0.68	1.6 [0.19–12.8]
Total scaffold length (mm)	0.25	1.0 [0.98–1.0]
Maximum inflation pressure scaffold deployment (atm)	0.89	1.0 [0.71–1.5]
Post-dilatation in all scaffolds (%)	0.72	1.3 [2.9]
Post-dilatation: Balloon diameter (mm)	0.38	3.6 [0.2–67]
Post-dilatation: Maximum inflation pressure (atm)	0.87	1.0 [0.5–1.9]

Table S6: Univariate analyse of OCT findings for primary endpoint (late or very late ScT).

	p	Hazard ratio
Number of struts	0.88	1.0 [0.99–1.0]
Number of frames	0.95	1.0 [0.98–1.0]
Pullback length (mm)	0.33	0.9 [0.80–1.1]
Maximum lumen area (mm^2)	0.009	1.2 [1.1–1.5]
Minimum lumen area (mm^2)	0.53	1.4 [1.0–2.1]
Average lumen area (mm^2)	0.008	1.5 [1.1–1.9]
Maximum scaffold eccentricity	0.03	0 [0–0.4]
PSLIA (%)	0.21	4.0 [0.45–36.2]
Microvessels (%)	0.42	1.9 [0.42–8.3]
Fractures with gap (%)	0.003	9.7 [2.2–43.6]
Uncovered scaffold struts (%)	0.003	9.8 [2.2–44.0]
Malapposition (>30% of the struts in at least one frame, %)	0.004	11.4 [2.2–58.8]
Any malapposition (%)	0.005	21.3 [2.6–177.1]
Malapposition length (mm)	0.47	0.8 [0.5–1.4]
Malapposition max area (mm^2)	0.28	0.73 [0.4–1.3]
Number of malapposed segments	0.94	1.03 [0.5–2.2]
Malapposition distance (mm)	0.21	0.26 [0.03–2.1]
Evaginations (%)	0.16	2.9 [0.65–13.1]

Table S7: Multivariable Cox regression analysis for primary endpoint (late or very late ScT).

	p	Odds ratio (95% CI)
Total scaffold length (mm)	0.67	1.0 [0.9–1.0]
Any malapposition (per patient)	0.02	13.7 [1.5–124.9]
Uncovered struts	0.05	4.9 [1.0–23.7]
Minimum lumen area (mm^2)	0.42	1.2 [0.8–1.8]
Fractures with gap	0.99	0 [0]
Number of vessels treated	0.06	17.8 [0.9–345.9]

Table S8: OCT findings depending on presence of malapposition.

	No malapposition (n = 153)	Malapposition (n = 44)	p
Number of struts	1105 ± 497	991 ± 499	0.15
Number of frames	121.8 ± 51.5	114.9 ± 48.4	0.64
Pullback length (mm)	20.9 ± 5.5	21.8 ± 5.9	0.25
Maximum lumen area (mm^2)	8.4 ± 2.5	11.6 ± 3.8	<0.001*
Minimum lumen area (mm^2)	4.6 ± 1.8	5.6 ± 2.1	0.008*
Average lumen area (mm^2)	6.3 ± 1.9	8.1 ± 2.5	<0.001*
Maximum lumen asymmetry	0.27 ± 0.11	0.30 ± 0.12	0.069
Maximum scaffold asymmetry	0.23 ± 0.09	0.26 ± 0.11	0.14
Maximum lumen eccentricity	0.73 ± 0.08	0.71 ± 0.09	0.17
Maximum scaffold eccentricity	0.68 ± 0.09	0.61 ± 0.12	<0.001*
PSLIA (%)	6.2	6.1	0.98
Microvessels (%)	31.3	31.8	0.95
Fractures (%)	33.8	36.4	0.75
Uncovered scaffold struts (%)	5.3	13.6	0.06
Evagination (%)	23.7	45.5	0.005

Table S9: Baseline characteristics of patients with ScT.

	Acute or subacute ScT (n = 7)	Late or very late ScT (n = 9)	p
Age (years)	59.1 ± 9.1	61.6 ± 7.4	0.68
Male sex (%)	57.1	0	0.03*
Hypertension (%)	71.4	88.9	0.38
Diabetes mellitus (%)	14.3	33.3	0.38
Current smoker (%)	28.6	33.3	0.83
Family history (%)	42.9	33.3	0.70
Hyperlipoproteinaemia (%)	57.1	22.2	0.15
Prior revascularization (%)	0	66.7	0.006*
Prior percutaneous intervention (%)	0	66.7	0.006*
Prior bypass surgery (%)	0	0	n.a.
Prior stroke/TIA (%)	0	0	n.a.
eGFR (mean±SD, ml/min)	63.1 ± 22.2	87.2 ± 24.9	0.09
Left ventricular ejection fraction (mean±SD, %)	47.5 ± 13.7	47.7 ± 11.8	0.95
Acute coronary syndrome (%)	85.7	77.8	0.69
Clinical indication			
Stable angina (%)	14.3	22.2	0.68
ST-elevation myocardial infarction (%)	28.6	33.3	0.84
Non-ST-elevation myocardial infarction (%)	28.6	44.4	0.52
Unstable angina (%)	28.6	0	0.09
Number of vessels treated per patient	1.0 ± 0	1.4 ± 0.7	0.30
Number of scaffolds per lesion	1.1 ± 0.38	1.6 ± 1.3	0.76
Number of scaffolds per patient	1.1 ± 0.4	2.1 ± 1.5	0.14
Chronic total occlusion (%)	0	11.1	0.36
Lesion type B2/C, %	57.1	77.8	0.37
Dual antiplatelet therapy (DAPT)			0.66
Clopidogrel (%)	28.6	11.1	
Prasugrel (%)	57.1	66.7	
Ticagrelor (%)	14.3	22.2	

Table S10: Procedural characteristics in the retrospective cohort of patients with ScT.

	Acute or subacute ScT (n = 7)	Late or very late ScT (n = 9)	p
Pre-dilatation (%)	85.7	100	0.24
Pre-dilatation: Balloon diameter (mm)	2.8 ± 0.3	2.9 ± 0.2	0.29
Minimum scaffold diameter per lesion (mm)	3.0 ± 0.3	3.1 ± 0.5	0.54
Minimum scaffold diameter per patient (mm)	3.0 ± 0.3	2.9 ± 0.5	0.46
Total scaffold length (mm)	21.3 ± 5.7	46.0 ± 38.8	0.41
Maximum inflation pressure scaffold deployment (atm)	14.6 ± 1.5	13.8 ± 1.2	0.29
Post-dilatation in all scaffolds (%)	57.1	55.6	0.95
Post-dilatation: Maximum inflation pressure (atm)	15.1 ± 1.9	14.3 ± 1.2	0.35
Overlap (%)	0	22.2	0.18

Table S11: OCT findings in the retrospective cohort (OCT at the time of ScT).

	Acute or subacute ScT (n = 7)	Late or very late ScT (n = 9)	p
Number of struts	116.2 ± 43.0	147.5 ± 103.1	0.66
Frames with thrombus, n	78.0 ± 36.6	55.0 ± 66.0	0.18
Malapposed struts, n	10.0 ± 24.5	9.6 ± 9.0	0.18
Malapposed frames, n	11.3 ± 27.7	20.1 ± 17.9	0.18
Uncovered struts, n	186.8 ± 415.5	0.13 ± 0.35	0.006*
PSLIA (% of patients)	16.7	50	0.20
Evagination (% of patients)	0	25.0	0.19
Malapposition (% of patients)	16.7	87.5	0.008*
Incomplete stent apposition area (mm^2)	1.5 ± 2.1	3.5 ± 1.9	0.67
ISA at maximum lumen (mm^2)	15.5 ± 0	10.9 ± 4.9	0.67
Fractures (% of patients)	16.7	75.0	0.03*
Minimum lumen area (mm^2)	2.6 ± 2.1	4.4 ± 3.6	0.35
Maximum lumen area (mm^2)	6.2 ± 3.3	9.5 ± 4.4	0.14
Maximum scaffold area (mm^2)	6.7 ± 2.7	11.1 ± 3.4	0.06
Minimum scaffold area (mm^2)	4.5 ± 2.8	5.8 ± 2.9	0.35
Incomplete expansion (% of patients)	83.3	11.1	0.008*
Length (mm)	20.2 ± 3.7	24.0 ± 19.1	0.54
Reference lumen area (mm^2)	7.1 ± 3.4	10.2 ± 2.6	0.05
Minimum diameter (mm)	2.1 ± 0.6	23 ± 0.8	0.66
Maximum diameter (mm)	3.0 ± 0.8	3.7 ± 0.9	0.14
Asymmetry index	0.3 ± 0.1	0.4 ± 0.2	0.49
Minimum eccentricity index	0.7 ± 0.1	0.7 ± 0.1	1.0
Neointima (% of patients)	0	37.5	0.09
Dismantling (% of patients)	0	44.4	0.04*

Percentages are expressed as % of the patients/scaffolds (some of the analyses were not possible in all segments due to the presence of thrombus).

Table S12: Dual antiplatelet regime in the cases in which OCT was diagnosed at the time of ScT.

N = 16	Acute or subacute scaffold thrombosis	Late or very late scaffold thrombosis
Clopidogrel	28.6	11.1
Prasugrel	57.1	66.7
Ticagrelor	14.3	22.2
